# Isothermal Storage Delays the Senescence of Post-Harvest Apple Fruit through the Regulation of Antioxidant Activity and Energy Metabolism

**DOI:** 10.3390/foods12091765

**Published:** 2023-04-24

**Authors:** Lan Chen, Mengya Wang, Haifen Wang, Cong Zhou, Junwei Yuan, Xihong Li, Yanfang Pan

**Affiliations:** 1 International Centre in Fundamental and Engineering Thermophysics, Tianjin University of Commerce, Tianjin 300134, China; chenlan890804@tjcu.edu.cn; 2 Institute of Food Science and Technology, Chinese Academic of Agricultural Sciences, Beijing 100193, China; 3 Shanxi Fruit Industry Cold Chain New Material Co., Ltd., Tongchuan 727100, China; 4 State Key Laboratory of Food Nutrition and Safety, College of Food Science and Engineering, Tianjin University of Science and Technology, Tianjin 300457, China; 13012422878@163.com (M.W.); haifenwang81@126.com (H.W.); zhoucong9704@163.com (C.Z.); junweiyuan@126.com (J.Y.); 5 Tianjin Gasin-DH Preservation Technologies Co., Ltd., Tianjin 300300, China

**Keywords:** apple fruit, isothermal storage, temperature fluctuation, senescence, antioxidant system, energy status

## Abstract

The purpose of this work was to elucidate the influence of TF (5 ± 5 °C, and 5 ± 1 °C) and CT (5 ± 0.1 °C served as an isothermal state) storage environment on the antioxidant ability and energy metabolism in post-harvest apple fruit during storage. Specifically, compared with fruit in TFs groups, the quality attributes of apples in the CT group, including firmness, fresh weight, contents of SSC, and TA were maintained at a higher level. In addition, fruit stored in the CT environment revealed a suppressed respiration rate and EL, lower MDA, O_2_·^−^, and H_2_O_2_ accumulation but increased the activities of SOD, CAT, APX, and GR. At the end of storage, the SOD, CAT, APX, and GR activities of fruit in the CT group were 38.14%,48.04%, 115.29%, and 34.85% higher than that of the TF5 group, respectively. Fruit in the CT environment also revealed higher AsA, GSH, total phenols, and total flavonoid content. In addition, fruit stored in the CT environment maintained higher ATP content, EC, and more active H^+^-ATPase, Ca^2+^-ATPase, CCO, and SDH. At the end of storage, the SDH and CCO activities of fruit in the TF0.1 group were 1.74, and 2.59 times higher than that in the TF5 group, respectively. Taken together, we attributed the fact that a constant temperature storage environment can retard the fruit senescence to the enhancement of antioxidant capacities and maintaining of higher energy status in apple fruit.

## 1. Introduction

Among the four major factors—temperature, humidity, gas, and anti-corrosion that determine the post-harvest quality of horticultural produce, the contribution rate of temperature can reach about 60% [1]. Owing to the fact that most of the physical, biochemical, microbiological, and physiological reactions contributing to the deterioration of fruit quality greatly depend on it, temperature plays an important role in determining the post-harvest quality of fresh agricultural products [2]. In plant physiology, biological reaction rates vary by 2–3 times for each 10 °C difference in temperature [3,4]. Therefore, storing the produce at a low and constant temperature (CT) that does not cause chilling injury has always been an efficient strategy to retain the post-harvest quality and prolong the shelf life of fresh agricultural products after harvest [5]. In fact, during the cold chain, from precooling, transportation, storage, distribution, and retail, until sold to consumers, the storage temperature environment for fresh agricultural products is constantly changing. Even in the storage stage, they are exposed to various temperature fluctuations (TFs) for many reasons, such as heat transfer of enclosure structure, defrosting of air coolers, goods incoming and outgoing, power outage, temporary equipment failure, and energy-saving strategy [6,7].

It is generally known that a highly humid environment, which is a favorable condition for fungus growth, will be triggered under TF conditions. This produces rot which ultimately increases levels of food wastage [8]. In addition, TFs also affect the physiological quality of horticultural produce. Although the present research on TF is mainly concentrated on the quality loss dynamics of frozen food during storage [7,9,10,11] and the energy savings strategy during storage of horticultural produce, and limited literature shows that it is detrimental to the storage quality of horticultural produce stored in the TF environment [6,12,13]. Kablan et al. found that the modified atmosphere packaging of mushrooms, broccoli, and mature green tomato were destroyed under un-isothermal conditions which triggered browning, softening, water loss, and decaying of fruit [14]. Xin et al. reported that the degradation of sodium carbonate-soluble pectin in the cell wall of sweet cherry was accelerated under non-isothermal conditions, ultimately leading to sweet cherry softening [15]. Peach exposed to higher TF (1 ± 2 °C) exhibited higher respiration and poorer soluble solids content compared to fruit in the CT environment (1 ± 0.1 °C) [16]. TF is detrimental to the storage quality of fresh agricultural products has been confirmed for apple [17], mature green tomato [18], strawberries [19], and table grapes [20,21]. All these reports provide evidence that the TF condition will accelerate the senescence of fresh horticultural produce, while the CT condition is helpful to maintain the quality of products. However, there is limited research on the physiological mechanisms of quality improvement and senescence delayed by the CT environment.

The senescence of fresh agricultural products is generally considered to be related to reactive oxygen species (ROS) burst events, when the production of enhanced ROS, such as the superoxide anion (O_2_·^−^), the hydrogen peroxide (H_2_O_2_), and hydroxyl radical (·OH), exceeds their degradation [22]. Normally, the production of ROS and the ability to scavenge them in tissue remains in a dynamic equilibrium state under the scavenging of enzymatic scavengers., examples of ascorbate peroxidase (APX), catalase (CAT), glutathione reductase (GR), superoxide dismutase (SOD), etc. and non-enzymatic scavengers, including ascorbic acid (AsA), glutathione (GSH), tocopherols, carotenoids, phenolics, etc. [23,24].

However, the scavenging capacity of these antioxidant systems decline when fruit undergoes senescence or is exposed to environmental stress (i.e., temperature, drought, high light, salinity, etc.). Then the accumulated ROS can target vital biomolecules (i.e., lipids, proteins, DNA, etc.) which trigger the disorder of membrane properties, cell physiological pathways, signaling cascades, and ultimately cause cell death [25,26]. Furthermore, increasing evidence supports the argument that the senescence of horticultural produce is closely linked to energy metabolism and that greater energy deficits are associated with more serious senescence [27,28,29]. Waning or inadequate availability of ATP induces ROS production and weakens antioxidant capacity, ultimately leading to membrane damage [30].

As one of the four major fruits (i.e., apple, grape, orange, and banana) in the world, apples are exposed to various levels of TFs during post-harvest circulation, including the storage process, and this inevitably causes food waste. To the best of our knowledge, the regulations of fruit senescence by the TF and CT environments in post-harvest apples are largely unknown. Furthermore, it is still unclear how TF and CT environments regulate the antioxidant ability and energy metabolism. With these issues in mind, the objective of this study was to evaluate the effects of TF and CT environments on the storage quality of apples and to investigate the senescence mechanisms involved. Here, the focus was on antioxidant response and energy charge. The results obtained in this study will help us to elucidate the importance of the CT environment in post-harvest storage for apple fruit from the perspective of fruit physiological metabolism as well as provide potential ideas to improve apple storage quality and economic value for producers and consumers.

## 2. Materials and Methods

### 2.1. Plant Materials and Treatments

**Figure 1:**
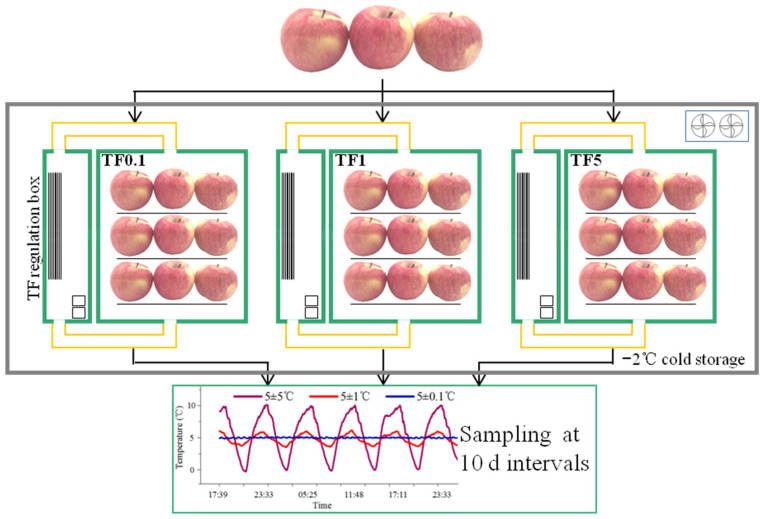
Schematics of TF0.1, TF1, and TF5 treatments. TF0.1, TF1, and TF5 indicated apples stored in 5 ± 0.1 °C, 5 ± 1 °C, and 5 ± 5 °C, respectively. The curve was the temperature monitoring of the TF regulation boxes in a certain experimental stage.

Apple fruit (cv. Fuji) at a commercial maturity (firmness of 84.5 ± 0.93 N, TSS of 15.7 ± 0.88%, and TA of 0.46 ± 0.02%) was harvested from Hongqipo Farm Gardening, Aksu City, Xinjiang, China (80°20′ E, 41°28′ N, Altitude 1105.3 m), packaged in cartons, and transported to the Agricultural Products Processing and Preservation Laboratory of Tianjin University of Science and Technology within 24 h. Fruit of uniform size and without disease or mechanical damage were selected as experimental materials, then randomly allocated into three groups of 447 fruit each, consisting of three replicates (149 fruit per replicate). As shown in Figure 1, the three groups were stored in the following temperature oscillations with 85–90% relative humidity for 60 d, respectively.

TF0.1: 5 ± 0.1 °C (4.9 °C→5 °C→5.1 °C, served as CT condition).

TF1: 5 ± 1 °C (4 °C→5 °C→6 °C).

TF5: 5 ± 5 °C (0 °C→5 °C→10 °C).

As illustrated in Figure 1, the TF environments were realized by creating three temperature gradient boxes. The structure and wording principle of the TF regulation boxes were described in our previous research [18]. During the entire experiment process, the gas concentration in the boxes was controlled by air composition.

The apple fruit was sampled at 10 d intervals during storage. At each sampling time, 20 fruits were taken from each replicate and assessed after being equilibrated to 20 ± 1 °C (overnight). Among them, 6 fruits were used for respiration rate, weightless, firmness, TSS, TA, and electrolyte leakage (EL) evaluation, and the 14 fruit left were cut into ~2 cm^2^ pieces, frozen in liquid nitrogen, and stored at −80 °C for subsequent experiments. All of the analyses stated above were carried out in triplicate. The experiment was conducted twice. However, because the experiments yielded similar results, data from only one of the experiments were presented.

### 2.2. Analysis of the Physical Parameters of Freshness

Respiration rate was measured with a respiration rate meter (HM-GX10, Shandong Hengmei Electronic Technology Co., Ltd., Weifang, China). It was measured as the amount of CO_2_ (mg CO_2_ kg^−1^ h^−1^). Weight loss was calculated by the gravimetric method and expressed as %. Firmness of 6 apples was determined with a digital penetrometer (GY-4, Zhejiang Top Instrument Co., Ltd., Hangzhou, China) with a needle-like probe of 10 mm diameter, and the result was expressed as N. The juice of 6 apples after crushing, homogenizing, and filtering was used to determine TSS and TA content. TSS was determined with a hand refractometer (DSA E-Scan, Electron Machine Corporation, Umatilla, FL, USA) and expressed as %. TA was measured by the neutralization titration method to pH 8.2 with 0.02 mol/L NaOH and expressed as a percentage of malic acid.

### 2.3. Analysis of Electrolyte Leakage (EL) and Malondialdehyde (MDA)

A total of 10 discs (10 mm diameter) from a 2 mm slice at the equator of 6 apples were washed with distilled water, dried with filter paper, and then used to determine EL according to the method employed by Cliff et al. [31]. Extraction and analysis of MDA content were conducted by the thiobarbituric acid reaction method according to the system described by Zhang et al. [32] and expressed as μmol g^−1^.

### 2.4. O_2_·^−^ Generation and H_2_O_2_ Content Assay

O_2_·^−^ generation rate and H_2_O_2_ content were assayed utilizing the method described by Li et al. [33] and Song et al. [34] and expressed as nmol min^−1^ g^−1^ and μmol g^−1^, respectively.

### 2.5. Analysis of the Activities of Antioxidant Enzymes

A total of 5 g of apple tissue was ground with 5 mL enzyme extraction buffer, and the ground tissue fluid was centrifuged at 11,000× *g* for 0.5 h at 4 °C. The supernatant was used for determining the enzyme activity. The extraction buffer of SOD and CAT was ice-cold phosphate buffer (0.1 M, pH 7.5) containing 5 mM DTT and 5% (*w*/*v*) PVP. The extraction buffer of APX was a potassium phosphate buffer (0.1 M, pH 7.5), containing 0.1 mM EDTA, 1 M ascorbic acid, and 2% (*w*/*v*) PVP. The extraction buffer of GR was an ice-cold phosphate buffer (0.1 M, pH 7.5) containing 0.1 mM EDTA.

SOD activity was determined using the method of Vicente et al. [35], and one SOD unit was defined as the enzyme amount that inhibits the reduction of NBT by 50% per minute. CAT activity was determined according to the method of Beers and Sizer [36], and one unit of CAT activity was defined as the change in absorbance with 0.01 at 240 nm per minute. The activity of APX was evaluated using the technique of Hu et al. [37]. One unit of APX was defined as the change of 0.01 absorbance at 290 nm per minute. GR activity was assayed according to the method of Ivan et al. [38]. One unit of GR activity was defined as the oxidation of 1 nmol NADPH per minute and the activity of all enzymes was expressed as U g^−1^.

### 2.6. Determination of Non-Enzymatic Antioxidants

AsA content was determined utilizing the method of Kampfenkel et al. [39] and expressed as mg 100 g^−1^. GSH content was assayed according to the system used by Ivan et al. [38] and expressed as μg g^−1^. The determination of total phenolic and total flavonoids was conducted as described by Pirie et al. [40] and calculated by gallic acid and rutin as standard curves, respectively, and expressed as mg g^−1^.

### 2.7. ATP, ADP, and AMP Contents and Energy Charge (EC) Measurements

The extraction and measurements of ATP, ADP, and AMP content were conducted following the methods described by Liu et al. [41] and expressed as μg g^−1^. EC was calculated by the formula: EC = (ATP + 1/2 ADP)/(ATP + ADP + AMP) × 100%.

### 2.8. Analysis of the Activities of Energy Metabolism Enzymes

Mitochondria were extracted as described by Jin et al. [30]. Succinic dehydrogenase (SDH) activity (U mg^−1^) was measured as reported by Wang et al. [42], and one unit of SDH activity was defined as an increase of 0.01 in absorbance per minute at 600 nm. Cytochrome C oxidase (CCO) activity (U mg^−1^) was measured as used by Jin et al. [43], and one unit of CCO activity was defined as an 0.01 change in absorbance per minute at 510 nm. H^+^-ATPase activity and Ca^2+^-ATPase activity were measured as reported by Jin et al. [44]. One unit of H^+^-ATPase (U mg^−1^) and Ca^2+^-ATPase (U mg^−1^) activity was expressed as the release of 1 μmol of phosphorus at 660 nm per minute, separately.

### 2.9. Statistical Analysis

All experimental data were obtained from three replicates from the three sub-samples. Data were expressed as the mean ± standard errors and analyzed with SPSS software (SPSS 19.0 for Windows, IBM, Armonk, NY, USA). The significance of the differences among samples was determined by Duncan’s multiple range tests in one-way analysis of variance (ANOVA) at the 0.05 level.

## 3. Results

### 3.1. Firmness, Weightless, TSS, TA and Respiration Rate of Apple

**Figure 2:**
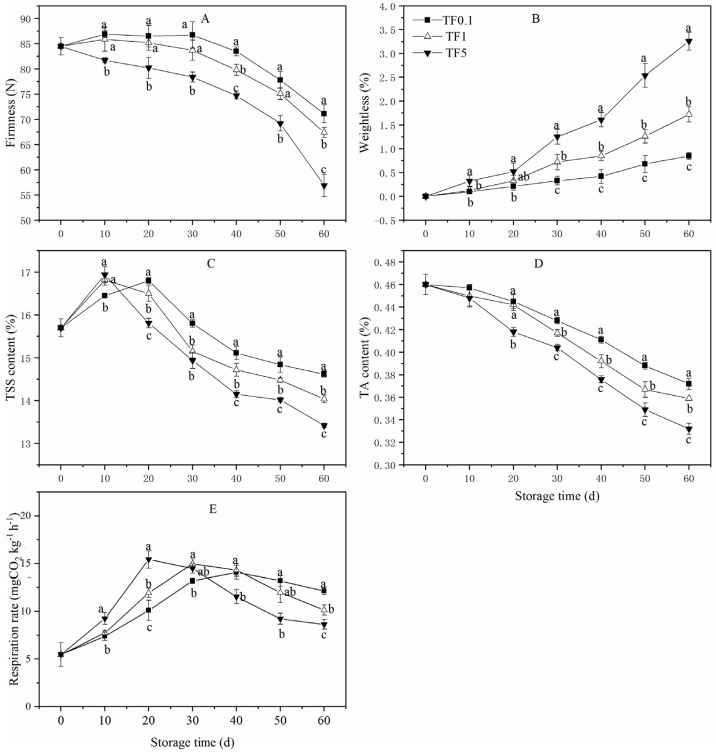
Firmness (**A**), weightlessness (**B**), TSS (**C**), TA (**D**), and Respiration rate (**E**) of apple fruit under different TF environments for 60 d. Different letters indicate significant differences among treatments (*p* < 0.05).

As depicted in Figure 2A, the firmness of apples under different TFs all showed a downward trend and declined slightly during the storage lasting 0–30 d, followed by a rapid decrease until the end of the storage period. During the entire storage period, apples subjected to TF5 retained firmness at a lower level with an overall decrease of 32.66%, while apples subjected to TF1 and TF0.1 decreased by 20.24% and 15.86%, respectively. When storage ended, the firmness of the three TF groups showed a significant difference (*p* < 0.05).

As shown in Figure 2B, the weightlessness of apples continuously increased during the whole storage period in each TF group. The increase was the slowest in the TF0.1 group, followed by the TF1 group, while that of TF5 group was the fastest. On storage day 60, the weight loss of TF0.1 group was only 26.07% of that concerning the TF5 group and 49.42% for the TF1 group.

In Figure 2C, the TSS content of TF0.1, TF1, and TF5 increased slightly at the early storage and then declined. The onset of TSS content decline in the TF0.1 group was delayed by 10 days compared to TF1 and TF5 groups. At the end of the storage period, the TSS content of the TF0.1 group was 14.62% which was 4.13% and 8.94% higher than that of the TF1 and TF5 groups, respectively (*p* < 0.05).

TA content (Figure 2D) in all TF groups indicated a similar decrease trend during the entire storage period with no significant difference during 0–10 d. However, the significant differences (*p* < 0.05) between the three groups were exhibited on the 40th day. Between 40 d and 60 d of storage, the TA content of the TF5 group fruit was, on average, 10.26% and 5.41% lower than that of the TF1 and TF0.1 groups of fruit, respectively.

The dynamic trends of the respiration rate of all fruit exhibited typical respiratory climacteric (Figure 2E). The respiration rate of fruit exposed to 5 ± 1 °C peaked at day 20, while those exposed to 5 ± 0.5 and 5 ± 0.1 °C TF were delayed for 10 days and 20 days, respectively. The respiratory of fruit in the TF0.1 group was at a relatively lower level in the early and middle storage periods but showed a higher level in the late stage due to the respiration decreasing in fruit in high TF groups.

### 3.2. EL, MDA Content, H_2_O_2_ Content, and O_2_·^−^ Generation Rate

**Figure 3:**
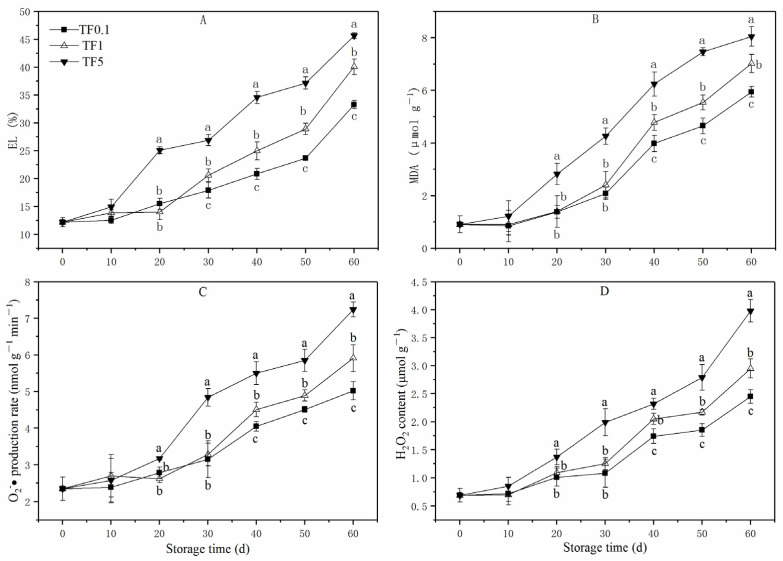
EL (**A**), MDA (**B**), O_2_·^−^ (**C**), and H_2_O_2_ (**D**) of apple fruit under different TF environment for 60 d. Different letters indicate significant differences among treatments (*p* < 0.05).

Figure 3A showed that the EL of all TF groups exhibited an upward trend during the whole storage period, and no significant differences were detected within the first 10 d. Afterward, the EL of apple in the TF5 group increased rapidly and was significantly higher than the TF1 and TF0.1 groups (*p* < 0.05) until the end. On the 60th day, the EL of the TF5 group was the highest which was 16.37% and 40.19% higher than that of the TF1 group and TF0.1 group, separately.

As displayed in Figure 3B, a similar increase in the MDA content of apple fruit was observed in all TFs. From the 10th day of the storage period, the MDA content in TF5 fruit showed an almost linear increase. During 60 d of storage, low TF conditions (TF1 and TF0.1) suppressed the accumulation of MDA. At the end of the storage, the MDA content of the TF5, TF1, and TF0.1 groups rose from the initial value of 0.91 μmol g^−1^ to 8.05 μmol g^−1^, 7.02 μmol g^−1^, and 5.94 μmol g^−1^, respectively. Remarkably the least MDA accumulation was observed in the case of the TF0.1 group.

The O_2_·^−^ production rate and H_2_O_2_ content in apple fruit increased continuously regardless of the given TF conditions (Figure 3C,D). Both the O_2_·^−^ generation and H_2_O_2_ content of fruit stored under TF5 condition increased quickly, while minimal TF conditions (TF1 and TF0.1, especially TF0.1) greatly diminished and delayed O_2_·^−^ generation and H_2_O_2_ accumulation. Furthermore, the H_2_O_2_ contents of the TF0.1 group were 16.95% and 38.44% lower than those in TF1 and TF5 groups on the 60th day of storage, respectively. It is also noted that the O_2_·^−^ production rates of the TF0.1 group were 15.06% and 30.66% lower than those in the TF1 and TF5 groups on the 60th day of storage, respectively.

### 3.3. Antioxidant Enzymes Activity

**Figure 4:**
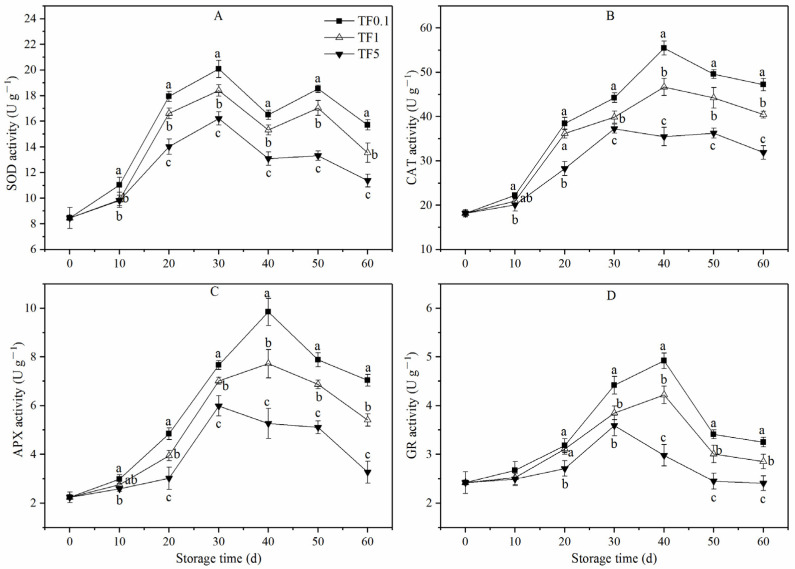
SOD (**A**), CAT (**B**), APX (**C**), and GR (**D**) activity of apple fruit under different TF environment for 60 d. Different letters indicate significant differences among treatments (*p* < 0.05).

As shown in Figure 4A, the SOD activity of apple fruits in all TF groups exhibited a similar trend during the storage period, i.e., a gradual increase in the early stage of storage peaked at the 30th day, then fluctuated downward. During the entire storage period, fruit stored under the TF0.1 condition showed a higher SOD activity than that under TF1 and TF5 conditions; of these, SOD activity under TF5 was the lowest. Taking the end of storage as an example, the SOD activity of the TF0.1 group was 15.72 U g^−1^ which was 16.10% and 38.14% higher than that of the TF1 and TF5 groups, respectively.

The changes in CAT, APX, and GR activities in apple fruit exhibited a similar trend during storage. As shown in Figure 4B–D, the activities of these three enzymes in the TF0.1 and TF1 groups did increase and reached the maximum on the 40th day, respectively, and then declined until the conclusion of the storage period. At the end of storage, the CAT activity of apple under the TF0.1 condition was 47.21 U g^−1^ which was 16.65% and 48.04% higher than that under the TF1 and TF5 conditions (*p* < 0.05). The APX activity was 30.13% and 115.29% higher than that under TF1 and TF5 conditions (*p* < 0.05) while GR activity was 22.64% and 34.85% higher than that under TF1 and TF5 conditions (*p* < 0.05). It could be inferred that CT condition was conducive to maintaining higher the SOD, CAT, APX, and GR enzyme activities of apple fruit.

### 3.4. Non-Enzymatic Antioxidants Content

**Figure 5:**
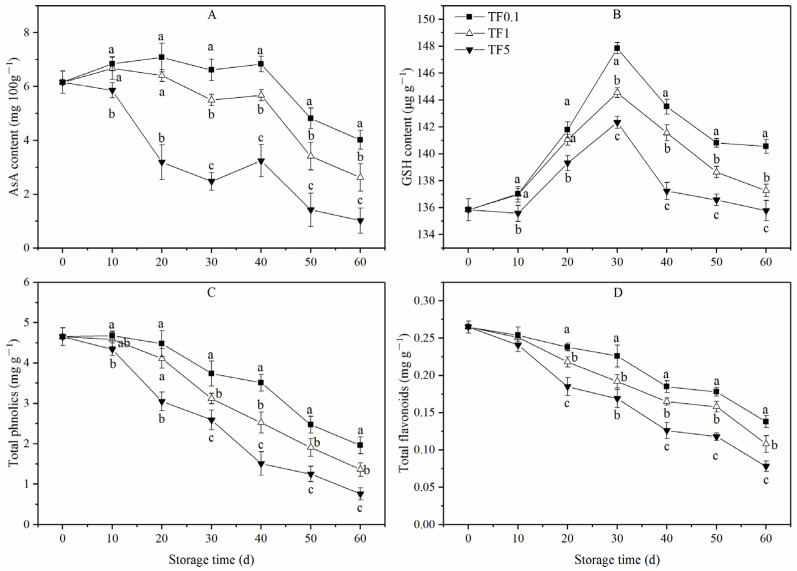
AsA (**A**), GSH (**B**), Total phnolics (**C**), and Total flavonolds (**D**) of apple fruit under different TF environment for 60 d. Values are the means ± SD (n = 3). Different letters indicate significant differences among treatments (*p* < 0.05).

The AsA content of all apples was on an overall downward trend (Figure 5A). Meanwhile, the low TF groups (TF1 and TF0.1) exhibited more AsA content than the TF5 group during the whole storage period (*p* < 0.05). Between 0 d and 40 d of storage, the AsA content of TF1 and TF0.1 altered slightly and, there after, decreased rapidly until the end of the storage. During the entire storage period, the TF0.1 group showed the highest AsA content with 4.02 mg 100 g^−1^ at the end of the storage which was 0.53 and 2.94 times higher than the TF1 group and TF5 group, separately.

The GSH content in all groups increased gradually to a peak value on day 30 and then declined (Figure 5B). For the TF5 group, the GSH content was significantly lower than that of the low TF groups (TF1 and TF0.1, *p* < 0.05), while that of the TF0.1 group was higher than the TF1 group from 20 d to 60 d. Significant differences were observed since day 30 (*p* < 0.05). On the 60th day of storage, the GSH content of the TF0.1 group was the highest with a value of 140.55 μg g^−1^ which was 2.38% and 3.51% higher than TF1 and TF5, respectively.

As demonstrated in Figure 5C,D, the total phnolics content and total flavonoids content of apple fruit exposed to all TF groups exhibited a similar declining trend as time passed. In comparison, the low TF conditions delayed the decline of total phnolics and total flavonoids to varying degrees. Among them, the TF0.1 condition displayed the most significant retention effect compared with the TF1 condition. Taking the total phenol content as an example for analysis, the total phenolics content of apple in the TF5 group fell from 4.65 mg g^−1^ on 0 d of storage to 0.76 mg g^−1^ on 60 d of storage, while apple exposed to TF0.1 condition dropped only to 1.96 mg g^−1^.

### 3.5. ATP, ADP, and AMP Contents and Energy Charge

**Figure 6:**
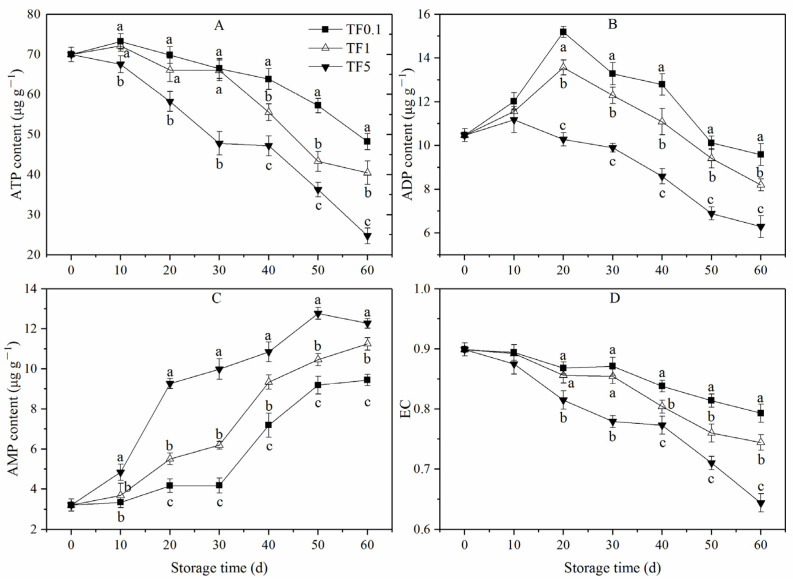
ATP (**A**), ADP (**B**), AMP (**C**), and EC (**D**) of apple fruit under different TF environment for 60 d. Different letters indicate significant differences among treatments (*p* < 0.05).

The ATP content of apple fruit exposed to low TF conditions (TF1 and TF0.1) exhibited a slight increase in the first 10 days and was followed by a gradual drop until the end of storage time, while that of the TF5 group steadily decreased during the entire storage period (Figure 6A). Apple fruit in TF5 conditions exhibited significantly less ATP compared to low TF conditions (*p* < 0.05). The ATP content of apples in the TF0.1 group was higher than that in the TF1 during late storage with significant differences observed at 40 d to 60 d (*p* < 0.05).

As shown in Figure 6B, the ADP content of fruit in the TF5 group rose slightly, peaked at 10 d, and then decreased until the end of the storage period, whereas that in TF1 and TF0.1 groups showed an obvious increase, peaked at 20 d, and exhibited a larger amount of ADP than that in TF5 from day 20 to day 60 (*p* < 0.05). In addition, fruit in TF0.1 group significantly (*p* < 0.05) maintained more ADP compared with fruit in the TF1 group from day 20 to day 60 (*p* < 0.05).

The AMP content of apple fruit in all TF groups gradually increased with the rise of storage time (Figure 6C). Among them, the AMP content of the TF0.1 group slightly increased during the storage period of 0 d to 30 d, and then increased rapidly from 30 d to 5 d, then tended to flatten. The TF1 and TF5 groups showed a more rapid increase in speed, especially the TF5 groups. The TF5 group exhibited a significantly larger amount of AMP than the low TF groups during the entire storage period (*p* < 0.05). In addition, the TF0.1 group showed the lowest AMP content compared with the TF1 group with significant differences observed at 90 d and 100 d (*p* <0.05).

The energy charge of all fruit gradually decreased with increasing storage time, especially fruit in the TF5 group (Figure 6D). As expected, the decline of the energy charge of apple fruit stored under low TF conditions, especially TF0.1, was suppressed. The energy charge of the TF1 and TF0.11 groups were about 6.59% and 23.14% higher than that in the TF5 group at the end of storage. High TF condition (TF5) tended to decrease the energy charge in the apple, while in contrast, the constant temperature (TF0.1) demonstrated a positive effect on the energy level of the apple.

### 3.6. Energy Metabolism Enzymes Activities

**Figure 7:**
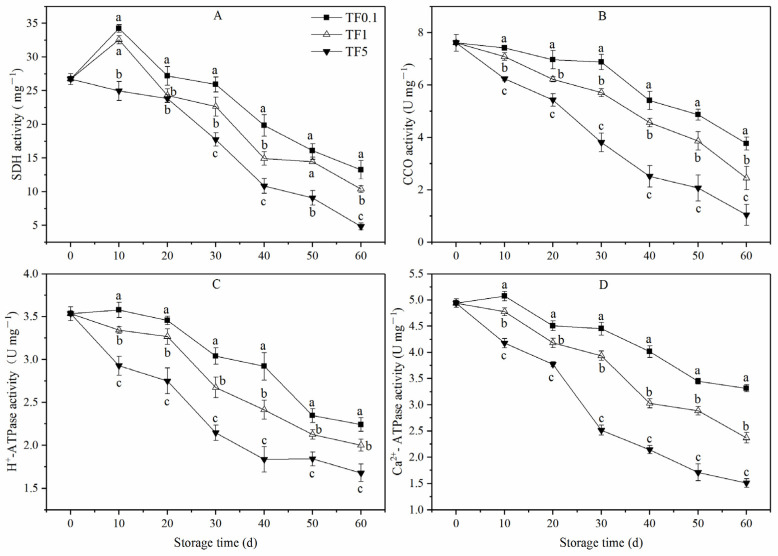
SDH (**A**), CCO (**B**), H+-ATPase, (**C**), and Ca^2+^-ATPase (**D**) activity of apple fruit under different TF environment for 60 d. Different letters indicate significant differences among treatments (*p* < 0.05).

In general, the SDH activity of apples stored in TF1 and TF0.1 conditions suffered an increase in the first 10 days, then decreased during the subsequent storage period, while that stored in the TF5 environment waned throughout the storage period (Figure 7A). Significantly (*p* < 0.05) lower SDH activity was observed in the TF5 fruit compared to the TF1 and TF0.1 fruit on day 30 of storage. Meanwhile, the SDH activity of fruit stored in TF0.1 was maintained at the highest level and was 0.27 and 1.74 times higher than in the TF1 and TF5 groups, respectively.

The CCO activity (Figure 7B) of all groups showed a similar trend that decreased with increasing storage time. The decline of the CCO activity in fruit in the TF0.1 condition effectively slowed down from day 0 to day 30, while that of fruit in the TF1 and TF5 conditions, especially in the latter, significantly accelerated. Fruit in the TF0.1 group exhibited a significantly higher CCO activity and was 0.54 and 2.59 times higher than that of the TF1 and TF5 fruit at the end of the storage phase, respectively.

In general, the activity of the H^+^-ATPase and Ca^2+^-ATPase decreased during the storage period (Figure 7C,D), except for slight increases found in the TF0.1 group on day 10. Fruit in the TF1 and TF0.1 groups exhibited higher H^+^-ATPase and Ca^2+^-ATPase activities than the TF5 group. Between 10 and 60 d of storage, the H^2+^-ATPase activity of fruit in the TF1 and TF0.1 groups were on average 15.85% and 26.39% higher than that in TF5 group, respectively (*p* < 0.05). Meanwhile, the Ca^2+^-ATPase activity of TF1 and TF0.1 groups were on average 17.51% and 43.27% higher than in the TF5 group (*p* < 0.05), respectively.

### 3.7. Pearson’s Correlation Coefficient Analysis

**Figure 8:**
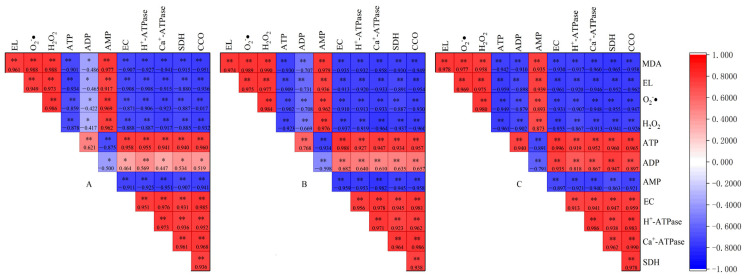
Pearson’s correlation coeffcients (*r*) among the analysis of membrane lipid peroxidation and energy metabolism indexes of apple fruit under 5 ± 0.1 °C (**A**), 5 ± 1 °C (**B**) and 5 ± 5 °C (**C**) during storage. Positive (Red) and negative (blue) correlations between analyses of respective values at * *p* < 0.05, ** *p* < 0.01. Numbers in the boxes represented the correlation coefficients.

To identify the relationship between membrane lipid peroxidation and energy metabolism of apples under different TFs during storage, Pearson’s correlation coefficient test (Figure 8) was undertaken. It revealed that the EC of the three TF groups was significantly positively correlated with the amounts of ATP and ADP, and the activities of SDH, CCO, H^+^-ATPase, and Ca^2+^-ATPase (*p* < 0.01) and was significantly negatively correlated with AMP (*r* = −0.907) (*p* < 0.01). This indicated that the energy state in apple cells was closely linked to the contents of ATP, ADP, and AMP and significantly affected by the key enzyme activities of mitochondrial respiration. Meanwhile, the EC of the three TF groups was significantly negatively correlated with EL, MDA, O_2_·^−^, and H_2_O_2_ (*p* < 0.01), indicating that maintaining higher energy levels of fruits played an important role in clearing ROS, such as O_2_·^−^ and H_2_O_2,_ in tissues. This reduced the degree of membrane lipid peroxidation and maintained membrane structural integrity.

In addition, as fluctuation in the temperature of the storage environment increased, the correlation between some indicators was enhanced, for example, the correlation between ADP and some indicators (such as EL, MDA, O_2_·^−^, H_2_O_2_, etc.) changed from significant (*p* < 0.05) to extremely significant (*p* < 0.01) (Figure 8A–C). It can be speculated that the process of the membrane lipid peroxidation of apples stored in the high TF environment was accelerated and aggravated which, in turn, caused severe oxidative stress in tissues, such as damage to the structure and function of mitochondria, which was extremely sensitive to ROS. Consequently, the activities of key respiratory metabolic enzymes on the inner mitochondrial membrane, specifically SDH, CCO, H^+^-ATPase, and Ca^2+^-ATPase decreased, leading to the energy dissipation and energy deficiency of tissue. Meanwhile, the energy deficiency of tissue might indirectly aggravate damage to the ROS scavenging system and the membrane damage caused by ROS attack, and finally accelerate the tissue senescence process. It should be noted that the constant storage temperature will delay the related process and, thus, delay the tissue senescence process.

## 4. Discussion

TF in the cold chain process can accelerate fruit senescence and quality deterioration, while CT storage can effectively maintain fruit quality and prolong storage. This has been reported in strawberries [19], tomatoes [18], Broccoli [14], etc. However, there are a few reports about the potential mechanism for the accelerated deterioration of fruit and vegetable quality and senescence caused by the TF of the storage environment.

The present work showed that low TF storage substantially suppressed the increase in weight loss and decrease in firmness and respiration rate in post-harvest apple fruit during storage time and delayed the arrival time of respiration peak. In addition, the suppression effect of the TF0.1 condition was more marked compared to the TF1 condition. Likewise, fruits, such as peaches [16] and apples [17], and vegetables, such as mushrooms, broccoli, and tomatoes [14], have been observed to be firmer and experience less weight loss when stored under low TF conditions. Besides, TSS and TA are important quality parameters of fruit, and as fruit senescence progresses, TSS and TA are consumed as respiratory metabolic substrates [45]. In this work, the higher level of TA and TSS in fruit in low TF conditions, especially fruit in the TF0.1 condition, were primarily caused by the reduced rate of respiration as shown in Figure 2E. This could be evidenced by the relatively low respiration of vegetables stored under CT compared to those under TF conditions which was reported by Kablan et al. [14].

The respiration process is one of the main sources of ROS [46]. The higher respiratory intensity (Figure 2E) was consistent with the higher ROS content of fruit in the TF5 group (Figure 3C,D). The oxidative stress caused by the surfeit of ROS accumulation accelerates the aging speed and reduces the storage quality of horticultural crops [46]. In this study, smaller ROS contents, including O_2_·^−^ and H_2_O_2,_ were noticed in apple fruit stored in the TF0.1 environment, suggesting that CT storage conditions were valid in reducing ROS stress. The accumulation of ROS also causes lipid peroxidation which leads to membrane deterioration and storage quality decreasing in fruit [46]. One recent work reported that the effective roles of CT storage in retarding fruit senescence are owed to its roles in reducing membrane lipid peroxidation stress [47]. Similar inhibition of lipid peroxidation stress associated with reduced fruit senescence of apples was also found in fruit stored in the CT environment in this study. As illustrated in Figure 3A,B, the CT condition (TF0.1) hindered the increase of EL and MDA content which further verified the apparent decrease of oxidative stress caused by CT storage. In all, the redox stress of apple fruit stored in the CT condition was attenuated and the membrane collapse caused by ROS stress was slowed down.

Free radical theory is one widely accepted theory for post-harvest fruit senescence. This theory holds that the decline of various metabolic activities and tissue senescence of post-harvest horticultural crops is the result of metabolic imbalance and the excessive accumulation of ROS [48]. As a response to ROS stresses, plants have evolved to a comprehensive antioxidant defense system to combat the harmful effects of ROS [49]. SOD can convert O_2_·^−^ into H_2_O_2_ which can be degraded into H_2_O and O_2_ by CAT. APX and GR reduce H_2_O_2_ to H_2_O through the GSH–AsA cycle [50]. All of them are well-known enzymatic antioxidants and are widely reported to be associated with fruit aging. In our study, the activities of CAT, APX, SOD, and GR of fruit in low TF groups (TF1, TF0.1), especially the TF0.1 group, were always maintained at a higher level compared to that in the TF5 group. There was a pronounced increase in the activities of SOD in peach fruit, and SOD and CAT in tomato fruit was also noticed when stored in the CT condition [16,18]. These results strongly suggest that the oxidative damage in apple fruits stored in the CT condition was reduced through the induction of antioxidant enzyme activities.

Besides antioxidant enzymes, AsA, GSH, phenols, and flavonoids are important components of the non-enzymatic ROS scavenging system in plants. AsA and GSH not only can directly reduce ROS by the AsA–GSH cycle but also play an important role in ROS scavenging as substrates of enzymes [51]. In this work, the contents of AsA and GSH in the TF5 group were always smaller than those in the TF1 and TF0.1 groups throughout the storage period. As well, the contents of these two antioxidants in the TF0.1 group were always significantly higher than the TF1 group in the middle and late storage period (*p* < 0.05). Furthermore, research has found that the high polyphenols and flavonoids content wield significant effects on the high antioxidant capacity and delay fruit senescence of apple [52,53]. In this study, the total phenolic and flavonoid content in apples tended to decline during storage and enabled the stability of the storage environment temperature to delay the decline of these two antioxidant components in apples. Meanwhile, high TF conditions will accelerate the decline. All these indicated that the non-enzymatic antioxidant defense system also played a pivotal role in the enhanced antioxidant capacity of apple fruit stored in constant temperature conditions.

Over recent years, studies have revealed that the aging of post-harvest fruit may be caused by low cellular energy production efficiency and insufficient energy supply [54,55,56]. Generally, ATP produced by mitochondria is the main energy source for life activities. SDH, CCO, Ca^2+^-ATPase, and H^+^-ATPase are the key enzymes involved in oxidative phosphorylation and ATP synthesis in plants [57]. Maintaining the activities of these enzymes consistently could provide sufficient energy for the cells’ survival under abiotic stress conditions and thereby improve storage tolerance in peaches [30] and bananas [58]. Under stress, changes in the number, morphology, or ultrastructure of mitochondria and membrane potentially lead to the destruction of the respiratory electron transport chain, resulting in the energy deficiency of fruit [56]. This explained the lower ATP content and EC level of fruit in the TF5 group (Figure 6). Additionally, the energy deficiency of fruit accelerated respiratory production (Figure 2E) which was consistent with previous studies where high energy deficiency induced an increase in respiration of postharvest fruit [59].

In addition, CT storage maintained higher activities of SDH, CCO, Ca^2+^-ATPase, and H^+^-ATPase. In addition, compared with the TF1 and TF5 groups, during storage, the fruit in the TF0.1 group kept higher levels of ATP, ADP, and EC but a lower level of AMP. Suggested here is that maintaining a higher energy level and activities of relative enzymes contributed to the improvement of apple fruit storage under TF stress.

More studies are showing that the senescence of post-harvest fruits is closely related to the collapse of cell membrane structures caused by insufficient cellular energy supply [60,61,62,63]. The fact that ATP deficiency results in more production of ROS in fruit indicates that ATP can regulate ROS production [64]. Importantly, it was reported that ATP treatment increased the activities of SOD, CAT, and APX to inhibit the accumulation of ROS [65]. As anticipated, our work reported that CT storage was conducive to maintaining ATP content and EC compared to the TF storage, in parallel with less MDA and ROS accumulation, less EL, and higher antioxidant enzyme activities in fruit stored at a constant temperature (TF0.1). Based on these findings, we put forward that the higher antioxidant ability and less ROS accumulation of apples in the TF0.1 group might be partly owed to the higher energy level caused by CT storage.

## 5. Conclusions

Our work described the advantageous effects of CT storage on delaying fruit senescence and quality deterioration in harvested apple fruit which was associated with the relief of the accumulation of H_2_O_2_, O_2_·^−^, and MDA. This was possible due to the maintenance of antioxidant capacity, including the increasing levels of non-enzymatic antioxidants (phenols, flavonoids, AsA, and GSH) and the enhancing of the antioxidant enzyme activities of SOD, CAT, APX, and GR, thereby reducing oxidative damage. Moreover, the higher activities of H^+^-ATPase, Ca^2+^-ATPase, CCO, and SDH, and the sufficient energy of apples in CT conditions was also the reason for delayed quality degradation and oxidative film damage. The appropriate energy supply of apples in the CT condition that was sourced from the higher activities of H^+^-ATPase, Ca^2+^-ATPase, CCO, and SDH also accounts for the delayed deterioration in quality, higher antioxidant capacity, and delayed oxidative membrane damage. Overall, the probable mechanism of how the TF condition accelerated the senescence of post-harvest apple fruit was involved in the regulation of antioxidant capacity and energy status. Furthermore, the indirect reduction of oxidative stress caused by sufficient energy supply led to the aggravated oxidative damage of the plasma membrane. Finally, these results confirmed the important role of CT storage in controlling the storage quality and delaying the senescence of horticultural produce. Overall, this study can provide a theoretical basis for the development of constant temperature storage technology, facilities for fruits and vegetables, and provides potential ideas to improve apple storage quality and economic value for producers and consumers.

## Data Availability

The data are available from the corresponding author.
